# Transylvania University

**Published:** 1841-07

**Authors:** 


### TRANSYLVANIA UNIVERSITY.

A short delay in the issuing of our last number enabled us barely to mention the election of a professor of the Theory and Practice of Medicine in this University, as the successor of Professor Smith, who has returned to Baltimore. Dr. Elisha Bartlet, of Lowell, Massachusetts, the new incumbent, is, no doubt, a man of talents and learning, who has claims to the confidence of the public of a higher order than that of standing, as the Dean informs us, at the head of the list of contributors to the American Journal of the Medical Sciences, when that list is *alphabetical.*

